# The Interrelationships between Cytokines and Schizophrenia: A Systematic Review

**DOI:** 10.3390/ijms25158477

**Published:** 2024-08-03

**Authors:** Haibing Lv, Meng Guo, Chuang Guo, Kuanjun He

**Affiliations:** 1 College of Life Sciences and Food Engineering, Inner Mongolia Minzu University, Tongliao 028000, China; lv5421213@163.com (H.L.); guochuang@imun.edu.cn (C.G.); 2 Finance Office, Inner Mongolia Minzu University, Tongliao 028000, China; dameng@imun.edu.cn

**Keywords:** schizophrenia, cytokines, biomarkers, extracellular vesicles, research advances

## Abstract

Schizophrenia (SCZ) imposes a significant burden on patients and their families because of its high prevalence rate and disabling nature. Given the lack of definitive conclusions regarding its pathogenesis, physicians heavily rely on patients’ subjective symptom descriptions for diagnosis because reliable diagnostic biomarkers are currently unavailable. The role of the inflammatory response in the pathogenesis of SCZ has been supported by some studies. The findings of these studies showed abnormal changes in the levels of inflammatory factors, such as cytokines (CKs), in both peripheral blood and cerebrospinal fluid (CSF) among individuals affected by SCZ. The findings imply that inflammatory factors could potentially function as risk indicators for the onset of SCZ. Consequently, researchers have directed their attention towards investigating the potential utility of CKs as viable biomarkers for diagnosing SCZ. Extracellular vesicles (EVs) containing disease-specific components exhibit remarkable stability and abundance, making them promising candidates for biomarker discovery across various diseases. CKs encapsulated within EVs secreted by immune cells offer valuable insights into disease progression. This review presents a comprehensive analysis summarizing the relationship between CKs and SCZ and emphasizes the vital role of CKs encapsulated within EVs in the pathogenesis and development of SCZ.

## 1. Introduction

Schizophrenia (SCZ) is a multifaceted and debilitating mental illness characterized by disturbances in cognition, perception, affect, and behavior [1,2]. The lifetime prevalence of SCZ is approximately 1%, with etiological factors including genetic and environmental influences [3,4]. While the precise etiology of SCZ remains elusive, emerging evidence suggests a complex interplay between persistent central nervous system (CNS) inflammation and the pathogenesis of SCZ [5]. Inflammatory processes can disrupt the delicate balance of neurotransmitters, such as dopamine and glutamate, which are known to play a crucial role in the manifestation of psychotic symptoms [6]. Inflammation may also induce oxidative stress and damage to neurons, affecting brain structure and function in individuals with SCZ [7]. In recent years, studies have identified evidence of elevated levels of inflammatory biomarkers in individuals with SCZ [8]. These markers encompass cytokines (CKs), which are small polypeptides or glycoproteins synthesized and secreted by multifarious tissue cells, mainly immune cells, in response to infection, injury, or stress. CKs can be classified into groups such as interleukins (ILs), interferons (INFs), and tumor necrosis factors (TNFs). These molecules serve as messengers, facilitating intercellular communication and performing diverse functions essential for maintaining overall bodily health [9]. By promoting inflammation, CKs aid in containing and eliminating threats to the body’s well-being [10]. Under certain circumstances, CKs can also exert neurotoxic effects by augmenting reactive oxygen species generation, thereby causing damage to neurons and other cells within the nervous system [11]. Moreover, CKs have the ability to modulate neurotransmitter transmission, consequently impacting intercellular communication among nerve cells [12]. These neurotoxic properties of CKs underscore the intricate interplay between the immune and nervous systems. In addition to their roles in immunology and inflammation, CKs play a pivotal role in regulating stress responses and adaptive behaviors [13]. During instances of injury or inflammation, immune-derived CKs can activate central stress-responsive neurotransmitter systems [14]. Research has reported changes in the levels of inflammatory CKs in individuals with SCZ compared to healthy individuals [15]. CKs such as IL-6, IL-1β, TNF-α, and IFN-γ have been observed to be elevated in the blood, CSF, and brain tissue of individuals with SCZ [16,17]. At same time, elevated levels of inflammatory CKs have been correlated with the severity of symptoms, cognitive impairment, and treatment resistance in SCZ [18,19]. The immune system plays a crucial part in the immunity against external stimuli and the maintenance of physiological homeostasis. 

Current research on mental illness is increasingly emphasizing the significance of immune system involvement. There is mounting evidence indicating that immune alterations associated with inflammation are intricately linked to an augmented susceptibility to SCZ. In prenatal investigations, pro-inflammatory CKs have been regarded as possible etiological factors responsible for the detrimental impact of infection on the developing fetal brain [20]. Furthermore, additional evidence suggests a correlation between increased levels of pro-inflammatory CKs, immune-related genes, and the manifestation of SCZ [21]. In the inflammatory response, there are pro-inflammatory CKs and anti-inflammatory CKs. Pro-inflammatory CKs can help activate many types of immune cells and promote inflammation. The involvement of pro-inflammatory CKs in the inflammatory process of the CNS is crucial, and dysregulated pro-inflammatory CKs can accelerate the development of SCZ when combined with genetic predisposition and glutaminergic neurotransmitters [17]. Anti-inflammatory CKs and other CKs may play a role in the occurrence and development of schizophrenia by activating certain cells and disrupting the inflammatory response [22].

Extracellular vesicles (EVs) are membranous bodies secreted by cells into the extracellular space and widely distributed in various bodily fluids. In 1983, EVs were found by Pan [23] et al. in sheep red blood cells. EVs facilitate the intercellular transfer of bioactive macromolecules, including DNA, proteins, mRNA, and non-coding RNA. The membranous vesicle structure of EVs confers protection to these molecules against degradation during long-distance transportation, thereby ensuring the preservation of their biological activity. The potential of EVs as non-invasive biomarkers for various diseases has garnered increasing interest in recent years. Due to their diverse range of inclusions and bidirectional ability to cross the brain–blood barrier (BBB), they can serve as a non-invasive biomarker for screening, diagnosing, and treating complex brain diseases. EVs have the capability to traverse the BBB and engage with immune receptors on glial cells, thereby inducing the production of CKs and inflammatory mediators, ultimately modulating brain function [24]. EVs and their inclusions are disease-specific, highly stable, and abundant, making them ideal biomarkers for a variety of diseases. Studying EVs and their inclusions can lead to a better comprehension of intercellular communication, disease initiation, and development. Currently, numerous studies have provided substantial evidence linking EVs and their inclusions to the initiation and progression of various diseases, thereby establishing their potential as disease-specific biomarkers for diagnostic purposes [25,26].

In this review, the relationship of CKs with SCZ was examined to understand the intricate interactions between the two. Understanding the complex interactions between inflammatory CKs and the central nervous system may provide valuable insights into the underlying mechanisms of SCZ, informing innovative therapeutic interventions to restore immune homeostasis and improve outcomes in patients with SCZ. 

## 2. Methods

This system evaluation is in accordance with the PRISMA guidelines for reporting, registration number for the doi: 10.37766/inplasy2024.7.0128. This review utilizes the PubMed database and Google Scholar to conduct a comprehensive search of articles published from 1996 to 2024. The most recent search was performed on 20 June 2024. Articles contained relevant keywords related to “schizophrenia”, “cytokine”, “biomarkers”, “anti-inflammatory therapy”, and “extracellular vesicles”. At least two authors meticulously examined the title and abstract of each article to determine its eligibility for full-text research. Only studies that could not be explicitly excluded based on the information provided in the title and abstract underwent further evaluation. Two additional reviewers assessed each remaining full-text article to ascertain its inclusion in this study. 

Inclusion criteria:

- Articles with an abstract available.
- Full text can be retrieved.
- Articles written in English.
- Species: human.

Exclusion criteria:

- Articles with incomplete or unavailable full text.
- Exclude non-English articles.
- Articles that lack significant relevance to this review.
- Exclude non-human studies.

**Figure 1:**
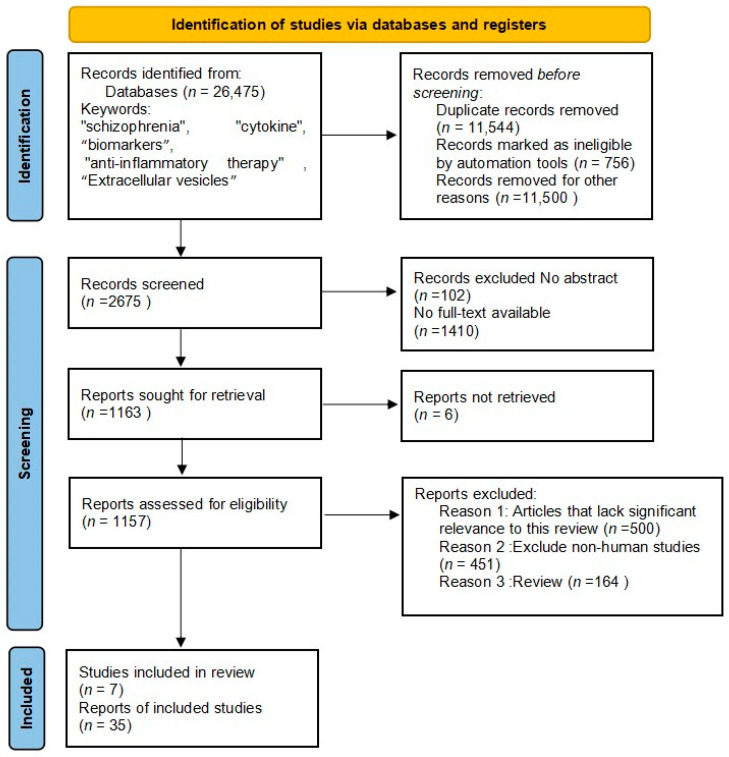
PRISMA flow chart.

The PRISMA flow chart is shown in Figure 1.

## 3. Results

Initially, we conducted a comprehensive search and identified a total of 2675 studies for screening purposes. Subsequently, articles without abstracts, those that were not searchable in full text, and non-English articles were excluded. The remaining 1157 articles were analyzed further by excluding 451 articles with non-human subjects and 500 articles that lack significant relevance to this review. Finally, after careful consideration, only forty-two articles were included in this review: eight meta-analyses; twenty-four original research articles; three clinical trials; and seven reviews.

### 3.1. Pro-Inflammatory Cytokines and Schizophrenia

Pro-inflammatory CKs can help activate many types of immune cells and promote inflammation. The involvement of pro-inflammatory CKs in the inflammatory process of the CNS is crucial, and dysregulated pro-inflammatory CKs can accelerate the development of SCZ when combined with genetic predisposition and glutaminergic neurotransmitters [17].

#### 3.1.1. IL-8

IL-8 expression was upregulated in patients with SCZ or in the first episode of psychosis (FEP) [27,28,29]. Brown et al. (2004) [30] demonstrated a positive correlation between elevated maternal serum levels of IL-8 during pregnancy and an increased susceptibility to SCZ in the offspring. Xu et al. (2018) [31] demonstrated a significant correlation between SCZ and IL-8 levels in peripheral blood mononuclear cells (PBMCs). Dahan [27] (2018) suggested that increased IL-8 levels in serum were associated with severe clinical symptoms of SCZ. Gallego et al. (2018) [28] confirmed a significant elevation in the IL-8 levels in CSF among patients with SCZ compared to controls. The meta-analysis confirmed an elevation of IL-8 level in the bloodstream of individuals with SCZ [32]. The findings demonstrated a consistent elevation of IL-8 concentrations in patients with both acute and chronic SCZ spectrum disorder [33]. These studies suggested that IL-8 perhaps plays a specific role in the pathophysiological alterations observed in individuals with SCZ. 

#### 3.1.2. IL-6

Some studies have shown elevated IL-6 levels in the serum or plasma of individuals with SCZ compared to healthy controls (HCs) [27,29,33,34,35,36,37,38,39]. Dahan et al. (2018) [27] suggested that elevated serum levels of IL-6 are related to severe clinical symptoms of SCZ. The expression level of IL-6 was found to be increased in FEP and acute relapse with SCZ, as well as SCZ patients who received initial treatment, when meta-analyses were employed to investigate CK expression in the plasma, serum, and CSF [15,28,40,41]. Notably, IL-6 levels decreased significantly in individuals with SCZ after treatment for acute illness or after treatment with antipsychotic medications [40,41]. Patlola et al. (2023) confirmed a significant association between elevated plasma levels of IL-6, IL-1β, CRP, and TNF-α and cognitive deficits in SCZ, thereby suggesting a potential association between inflammation and cognitive decline within this population [39]. Further research into the potential pathway between IL-6 may accelerate the discovery of ways to address disease progression in SCZ. However, these studies have limitations, such as the utilization of small sample sizes and the potential influence of confounding factors on IL-6 expression levels.

#### 3.1.3. IL-1β

There were five studies that showed that IL-1β was expressed at higher levels in the serum, plasma, or CSF of individuals with SCZ or FEP than in HCs [28,29,33,37,42]. Two meta-analyses showed that serum IL-1β levels were significantly elevated in SCZ patients with FEP and acute relapse when the study samples were derived from blood and CSF or serum CKs [15,40]. A meta-analysis revealed a significant reduction in the levels of IL-1β in patients undergoing antipsychotic treatment [40]. In addition, studies have shown that the presentation of psychotic symptoms in SCZ is positively correlated with IL-1β levels [37]. In this analysis, the possibility of random error is controlled, increasing the confidence in the elevated CK levels detected in the FEP. The findings imply a potential correlation between elevated IL-1β and the etiology of SCZ. Although these findings provide evidence for pro-inflammatory effects in SCZ, there is still heterogeneity among patient populations. The present studies possess certain limitations, as the outcomes are constrained by a limited sample size and potential confounding factors. Future investigations should aim to augment the sample size in order to comprehensively mitigate other plausible variables.

#### 3.1.4. TNF-α

Studies have shown that the expression of TNF-α is upregulated in the serum of individuals with SCZ [15,29,33,35]. However, one study concluded that serum TNF-α levels were significantly reduced in chronic SCZ [43]. This inconsistent structure may be attributed to the limited sample range and the different onset periods of the samples. Three meta-analyses of TNF-α expression levels in the blood, CSF, and serum of individuals with SCZ showed elevated TNF-α levels in individuals with SCZ [15,40,41]. Zhou et al. (2019) [44] compared the plasma level of brain-derived neurotrophic factor (BDNF) and other CKs in individuals with SCZ and HCs found that the plasma level of BDNF was negatively correlated with TNF-α in individuals with SCZ. Chukaew et al. (2022) [45] found an inverse correlation between serum TNF-α levels and S100B and the processing speed and attention in individuals with SCZ. Studies have controlled for the possibility of random error, increasing the confidence in elevated CK levels detected in FEP. Because CKs are usually regulated in a cascade fashion, there are some studies that may provide only partial insights into SCZ immune dysfunction using TNF-α alone. One limitation of these studies is that the small sample may not accurately represent prevailing trends in SCZ.

#### 3.1.5. IL-17

Dimitrov [46] et al. (2013) reported a significant reduction in the levels of IL-17 in SCZ [46]. The results suggest that dysregulation in the IL-17 pathway may contribute to the manifestation of psychotic symptoms in SCZ. Based on the findings of this study, further investigation into the IL-17 pathway is recommended to elucidate the underlying mechanism of SCZ symptoms. El Kissi et al. (2015) [47] revealed that the serum IL-17 level was significantly elevated in acute SCZ, while also observing a negative correlation between the overall SANS score and IL-17. Fang et al. (2018) [48] found no prominent difference in the blood levels of IL-17 between individuals experiencing first-onset psychosis and HCs. Li et al. (2016) [49] investigated the association between immune factors and revealed significantly elevated levels of plasma IL-17 in individuals with SCZ compared to HCs [49]. The inconsistencies in the results of these studies may be due to different patient periods selected, different sample sources collected, or different regions where the sampled individuals lived.

#### 3.1.6. IL-12

A meta-analysis revealed increased levels of IL-12 in patients with SCZ experiencing FEP and acute relapse with SCZ when analyzing blood and CSF CKs [40]. The research showed that IL-12 levels remained elevated in patients treated with antipsychotics [40]. These results suggest that antipsychotic drugs do not have an effect on IL-12 expression levels. The percentage of whole gray matter in SCZ exhibited a negative correlation with levels of IL-12 [37]. Furthermore, there was an observed trend between prefrontal cortex thickness and levels of IL-12 [37]. The findings suggest that elevated pro-inflammatory CKs may be associated with the etiology of SCZ, based on evidence of total gray matter reduction associated with CKs. The studies had certain limitations, as the sex ratios of the SCZ and control groups were not perfectly matched.

#### 3.1.7. IFN-γ

A meta-analysis revealed increased levels of IFN-γ in individuals with SCZ experiencing FEP and acute relapse with SCZ when analyzing blood and CSF CKs [40]. IFN-γ levels remained elevated in patients treated with antipsychotics [40]. Dimitrov [46] et al. (2013) revealed a significant reduction in the level of IFN-γ. [46]. The plasma IFN-γ level was elevated in individuals with SCZ compared to HCs [37]. The percentage of whole gray matter in SCZ exhibited a negative correlation with the IFN-γ level [37]. Furthermore, there was an observed trend between prefrontal cortex thickness and the level of IFN-γ [37]. The sex ratios of the SCZ and control groups did not precisely align, which poses limitations to the studies. The levels of IFN-γ exhibited a significant increase in individuals with acute SCZ spectrum disorders, whereas the levels of IFN-γ showed a significant decrease in those with chronic SCZ spectrum disorders [33]. This study could help to understand whether inflammatory biomarkers contribute to the diagnosis and prognosis of SCZ spectrum disorders. Further studies are needed to ascertain whether these peripheral changes are mirrored in the CNS. 

#### 3.1.8. IL-2

Two studies showed a significant downregulation of IL-2 in the peripheral white blood cells and serum of patients with SCZ [34,46]. However, two other studies reported significantly higher IL-2 levels in patients with SCZ than in controls [36,37]. The reason for this result may be that the sample selection is different, or the CK expression may be different in people living in different regions. Meanwhile, it should be noted that the sample size in these studies is relatively limited, and future investigations ought to consider expanding upon this limitation. 

#### 3.1.9. IL-23

A study revealed significantly elevated level of plasma IL-23 in patients with SCZ compared to HCs [49]. The serum IL-23 level was found to be significantly elevated in patients experiencing their FEP as well as those with recurrent SCZ, when compared to HCs [50]. Furthermore, it was observed that even after four weeks of antipsychotic treatment, the IL-23 levels remained persistently high in both patient groups [50]. The exploratory study establishes a fundamental basis for future research in the field by investigating the potential role of IL-23 in SCZ. The influence of other factors, such as BMI, gender, and smoking, should be acknowledged and considered in future analyses to ensure a comprehensive understanding of the results. 

#### 3.1.10. Chemokines

Dimitrov et al. (2013) [46] reported that patients with SCZ displayed significantly elevated serum sample levels of GRO, macrophage-derived chemokine (MDC), monocyte chemoattractant protein-1 (MCP-1), and sCD40L compared to HCs. Hong [51] et al. found that the plasma levels of MCP-1, categorization protein-11(CCL11), macrophage inflammatory protein-1β (MIP-1β), thymus- and activation-regulated chemokine (TARC), and MDC were elevated in individuals with SCZ compared to HCs. The study did not offer direct evidence regarding the involvement of chemokines in the pathophysiology of SCZ. Future research needs to evaluate whether treatment aimed at normalizing chemokine dysregulation can improve chronic SCZ.

The presence of a correlation between pro-inflammatory CKs and SCZ has been observed. The above related studies are shown in Table 1.

### 3.2. Anti-Inflammatory Cytokines and Schizophrenia

Currently, the investigation of anti-inflammatory CKs in SCZ is relatively limited compared to that of pro-inflammatory CKs. A cross-sectional study showed elevated IL-1RA in vitro in SCZ [34]. Kunz et al. (2011) showed that the anti-inflammatory factor serum IL-10 level in individuals with SCZ was elevated [52]. The findings offer support for the study of CKs as possible biomarkers of disease viability. The limitations of this study include confounding factors and the potential impact of antipsychotic drugs on CK outcomes. Xiu et al. (2014) [53] revealed a significant decrease in serum IL-10 levels in individuals with SCZ compared to HCs. However, Fu et al. (2019) [54] showed higher peripheral IL-10 in individuals with SCZ compared to HCs. The variations in the findings of these studies may be ascribed to factors such as the number of participants selected. Li et al. (2016) [49] revealed a significant elevation in plasma TGF-β1 levels among individuals with SCZ compared to HCs. Goldsmith et al. (2016) [41] conducted a meta-analysis of blood CKs in SCZ and observed that individuals with SCZ exhibited significantly increased IL-1 receptor antagonist (IL-1RA) levels. Zhou et al. (2019) [44] comparing the plasma level of BDNF and other CKs in individuals with SCZ and HCs revealed that individuals with SCZ had significantly elevated IL-1Ra levels compared to HCs. The majority of CKs demonstrated a positive correlation with positive, general, and total PANSS scores in individuals diagnosed with SCZ for a duration of 10 years or more [55]. Additionally, patients with a longer disease duration demonstrated higher serum IL-10 levels compared to those with a shorter duration of 5 years or less [55]. The limitations of this study include the inability to exclude the impact of antipsychotics on trial CKs and assess their effect on the study parameters. Halstead et al.’s (2023) [33] analysis found that IL-10 and IL-1RA concentrations continued to increase in patients with SCZ, while IL-4 concentrations continued to increase in patients with chronic SCZ spectrum disorder. The presence of a correlation between anti-inflammatory CKs and SCZ has been observed. The above related studies are shown in Table 2.

### 3.3. Other Cytokines and Schizophrenia

Bresee [56] et al. (2009) showed a persistently elevated serum sIL-2R level in individuals with SCZ compared to HCs. The results suggested that serum sIL-2R levels could potentially function as a biomarker of SCZ phenotype. The study was limited to reporting data on only one biomarker and the effects of drugs on sIL-2R. Xiu et al. (2015) [57] found that serum IL-3 levels in individuals with SCZ are significantly higher than in HCs. The correlation analysis revealed a statistically significant positive association between the level of IL-3 and the PANSS [57]. There are limitations to the study, including a relatively small sample and the potential for false positive or negative results. Additionally, confounding factors have an impact on CK levels. The underlying mechanism through which antipsychotic drugs modulate CKs warrants further investigation. Fu et al. (2016) also found that the serum levels of IL-3 in individuals with chronic SCZ were significantly elevated compared to those in HCs [58]. Marijuana is a widely used illicit drug, and the harmful effects on mental health have been widely reported, such as schizophrenic episodes [59]. Fernandez-Egea et al. (2013) indicated that marijuana usage is associated with an elevation in plasma levels of CCL11 [60]. The neurotransmitter system is directly regulated by cannabinoid receptors (CBRs) in the brain. In peripheral lymphocytes, CBRs mediate CK release, and individuals with SCZ exhibit dysregulated CK levels [61]. The presence of a correlation between other CKs and SCZ has been observed. The above related studies are shown in Table 3.

### 3.4. Anti-Inflammatory Treatment

SCZ patients frequently exhibit immune abnormalities leading to elevated CK levels. The emergence of immunomodulation as a potential therapeutic approach has given rise to novel strategies for treating SCZ [62,63]. Some medications with anti-inflammatory effects have some beneficial effects on SCZ symptoms [63]. The consumption of certain foods or adherence to specific dietary patterns can have either pro-inflammatory or anti-inflammatory effects [64]. Elevated levels of pro-inflammatory CKs and microglia activation have been observed in SCZ, which has been implicated in the pathogenesis of this disorder [17]. Omega-3 fatty acids are frequently recommended for individuals with SCZ due to their potent anti-inflammatory properties [65]. The coexistence of insomnia and inflammation is commonly observed in individuals diagnosed with SCZ. Among the broader populace, insomnia has been found to be associated with inflammation [66]. The sphingosine-1-phosphate receptor modulator fingolimod exhibits neuroprotective and anti-inflammatory properties [67]. Moreover, as an adjuvant therapy for SCZ, fingolimod demonstrates both safety and efficacy [67]. The tolerability of berberine treatment in SCZ patients has been demonstrated by multiple studies, and its efficacy in improving negative symptoms through anti-inflammatory mechanisms has been established [68]. Salehi et al. (2022) demonstrated the adjunctive administration of palmitoyl ethanol amide (PEA) and risperidone to be effective in alleviating primary negative symptoms associated with SCZ [69]. Risperidone treatment has been found to be related to alterations in serum pro-inflammatory CK levels and body weight, as reported by Song et al. (2014) [70]. The initial anti-inflammatory effect diminished during the course of treatment, exhibiting a significant decrease in the serum levels of IL-6, IL-1β, and TNF-α. This decline may be attributed to an associated side effect of weight gain, leading to a convergence of CK levels back towards baseline. Motamed’s (2022) [71] study demonstrated the efficacy of adjunctive therapy with adalimumab in the treatment of SCZ, particularly in alleviating negative and general psychopathological symptoms, while exhibiting no adverse effects. After 8 weeks of treatment, the levels of CRP, IL -1β, TNF-α, IL-8, and IL-6 decreased significantly [71]. 

Long et al. (2023) demonstrate a robust anti-inflammatory and neuroprotective role of minocycline and risperidone, elucidating the potential mechanism underlying their efficacy in treating negative symptoms in SCZ [72]. Minocycline significantly attenuated the production of IL-6, TNF-α, and IL-1β. Similarly, risperidone exhibited significant reductions in the levels of TNF-α and IL-6 [72]. A study revealed that clozapine exerts a more pronounced impact on immune function in female patients diagnosed with SCZ, as evidenced by lower serum IL-2 levels observed in the female cohort than in their male counterparts [73]. Interestingly, a significant positive correlation was observed between serum IL-2 levels and daily clozapine doses in female patients [73]. While recent analyses have advanced the field, further study is required to explain the relationship between antipsychotic drugs and CK levels. The above findings are summarized in Table 4.

### 3.5. The CKs in EVs and SCZ

One study enriched plasma- and neuron-derived EVs from FEP patients and matched controls [74]. The levels of leukemia inhibitory factor in astrocyte-derived EVs were found to be significantly lower in FEP patients compared to controls. Conversely, elevated levels of IL-6 have been observed in both plasma and brain tissue in various neurological disorders [74]. The EVs containing CKs and chemokine receptors undergo internalization, following which the internalized receptors transit to early endosomes and rapidly return to the plasma membrane. Within the plasma membrane, EVs interact with extrasynaptic and intrasynaptic N-methyl-D-aspartate receptors (NMDARs) and dopamine receptors (DARs), implying a potential association with SCZ [75,76].

## 4. Discussion 

This review provides a comprehensive analysis of the complex interplay between cytokine dysregulation and SCZ, emphasizing the bidirectional relationship between CKs and the disorder. Studies consistently show that patients with SCZ have significantly higher levels of pro-inflammatory CKs, such as IL-6 [15,27,28,29,33,34,35,36,37,38,39,40,41], TNF-α [15,29,33,35,38,39,40,41], and IL-1β [28,29,33,37,39,42], etc., in the blood or CSF compared to HCs. Notably, anti-inflammatory CKs also play a role in the occurrence and development process of SCZ [33,34,41,44,49,52,53,54,55]. Moreover, disturbances in cytokine profiles may precede the clinical onset of SCZ, indicating their potential as predictive biomarkers for the disease [77]. This review explores the impact of antipsychotic medication on cytokine levels, revealing a complex picture: some treatments may correct cytokine imbalances [68,70,71,72,73], while others could worsen them [78]. This underscores the need for a nuanced understanding of cytokine dynamics in the context of SCZ treatment. Additionally, the emergence of immunomodulatory therapies offers new opportunities for managing SCZ, potentially leading to significant treatment advancements.

**Figure 2:**
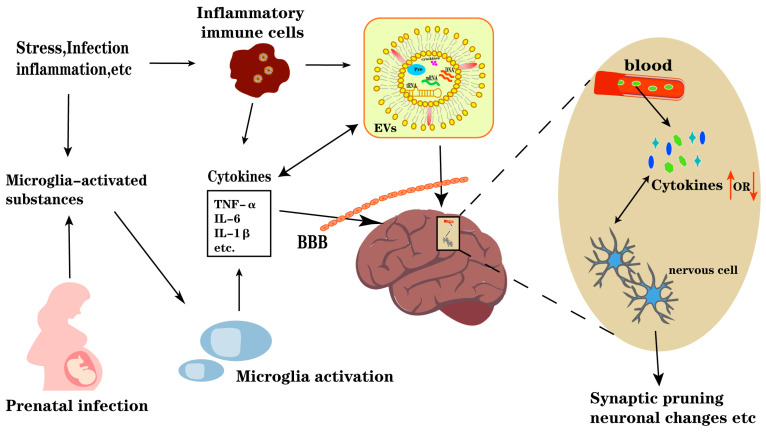
The potential mechanism diagram of cytokines participating in the brain inflammatory response. Abbreviations: BBB, brain–blood barrier; EVs, extracellular vesicles; CKs, cytokines. Red arrows: the up-regulation and down-regulation of cytokines. Bidirectional relationship: CK and cell can influence each other. One-way arrow: Unidirectional impact or generation of CK or CK into the cell.

This review synthesizes a multifaceted perspective on how immune system irregularities can contribute to neuropsychiatric disturbances, affecting synaptic plasticity, neurotransmitter systems, and blood–brain barrier permeability. It elucidates mechanisms by which CKs impact neuronal function and integrity, particularly through EVs. All immune cell types involved in inflammation are capable of secreting EVs, and EVs have multiple roles in the inflammatory process [79]. CKs in EVs secreted by immune cells are more reflective of the state of the disease. CKs and other circulating inflammatory mediators can traverse various pathways to access and exert their influence on the brain. These include leaky periventricular regions directly through the BBB, the lymphatic system, and indirect pathways [80]. EVs are involved in several physiological mechanisms closely related to SCZ, including cellular communication [81]. The EVs derived from mesenchymal stem cells (MSC-exo) facilitate neurogenesis and promote neuronal recovery by delivering trophic factors, vasoactive factors, and immunomodulatory factors to injured microglia and neurons while suppressing detrimental immune responses [82]. Tsivion-Visbord [83] et al. (2020) have shown that MSC-derived EVs improve SCZ-like behavior in mouse models of SCZ. By utilizing in vitro cultures for cell-type- and disease-specific characterization, the researchers established an exosome model system and observed a significant accumulation of specific EV epitopes in certain cell types of SCZ neurons (in comparison to healthy neurons) [84]. Life, inflammation, pregnancy, infection, and other factors can induce microglia to activate and produce CKs. Immune cells and the EVs they secrete can also secrete CKs. CKs have the ability to cross directly through the brain–blood barrier (BBB), which causes a series of changes in the brain. In addition, CKs can enter EVs secreted by inflammatory immune cells. Notably, these EVs have the ability to autonomously cross the BBB and have an impact on brain function. Thus, this cascade ultimately leads to the manifestation of mental illness while disrupting the systemic balance of CKs. Combined with previous studies, a comprehensive overview of CKs participating in the brain inflammatory response can be seen in Figure 2.

Despite the strengths of this review, several limitations must be acknowledged. The reliance on peripheral blood cytokine measurements may not accurately reflect CNS cytokine activity due to the blood–brain barrier. Sample size limitations and inadequate control for confounding factors such as smoking status, sex, age, and BMI could influence cytokine levels and affect the generalizability of findings. The impact of antipsychotic medications on cytokine levels also needs careful consideration, as these drugs can lead to misleading positive or negative results. Additionally, the correlative nature of the data makes it challenging to establish causality between cytokine dysregulation and SCZ.

Future research should aim to elucidate the direct impact of CNS-specific cytokine alterations and their interactions with neural circuits implicated in SCZ. Advanced imaging techniques combined with cerebrospinal fluid analysis could provide deeper insights into brain-specific cytokine profiles. Longitudinal studies with larger and more diverse populations are essential to validate cytokine biomarkers and explore their potential in predicting disease onset and treatment outcomes. Emphasizing the development of cytokine-modulating therapies and personalized medicine approaches could revolutionize SCZ treatment, addressing current gaps in the efficacy and side-effect profiles of existing antipsychotics. A comprehensive exploration of CKs that are intricately involved in the etiology of mental disorders is imperative.

## 5. Conclusions

Although some CKs in serum are associated with the development of SCZ and are supported by experimental validation results, there is no clear indication that CKs are responsible for the pathogenesis of SCZ. Inflammation may be one of the triggers, and CKs hold promise as a viable biomarker for the prevention and diagnosis of SCZ. Treatment with some anti-inflammatory medicines is also effective, and there are also studies that conduct treatment with anti-inflammatory drugs that may be more effective when combined with an anti-inflammatory diet. Considering the inherent characteristics and functionalities of EVs, it is inferred that CKs derived from EVs in immune cells may exhibit enhanced efficacy, thereby offering novel avenues for subsequent researchers to explore the diagnosis and treatment of SCZ. 

## Figures and Tables

**Table 1:** Summary of the studies that link schizophrenia and pro-inflammatory cytokines.

Authors, Year and Country	Tissue	Upregulated	Downregulated	Main Finding	Reference(s)
Potvin, S. et al., 2008, Canada	Serum plasma	sIL-2R, IL-1RA, and IL-6	IL-2	It provides evidence for the hypothesis of SCZ inflammatory syndrome.	[34]
Söderlund, J. et al., 2009, Sweden	CSF	IL-1β		This study provides evidence to support the activation of the brain’s immune system during the FEP.	[42]
Miller, B. J. et al., 2011, USA	Blood and CSF	IL-12, IL-6, IL-8, IFN-γ, TGF-β, IL-1RA, and TNF-α		The findings indicate that alterations in CK levels in SCZ may exhibit variability based on clinical status.	[40]
Dimitrov, D. H. et al., 2013, USA	Serum	GRO, MCP-1, MDC, and sCD40L	IFN-γ IL-2 IL-17	The levels of CKs in individuals with SCZ demonstrated a positive correlation with both positive symptoms and PANSS. Pathway analysis revealed the involvement of these CKs in the IL17 pathway, suggesting a potential role for this pathway in the progression of SCZ.	[46]
Di Nicola, M. et al., 2013, UK	Serum	IL-6, IL-1β, IL-8, and TNF-α		A history of childhood trauma was found to be related to elevated levels of serum TNF-α, while stressful life events were linked to increased expression of TNF-α mRNA in white blood cells. In summary, individuals experiencing their first episode of psychosis exhibit a pro-inflammatory state.	[29]
	Leukocyte	IL-6 and TNF-α			
Upthegrove, R. et al., 2014, UK	Plasma or serum	TNF-α, IL-1β, and IL-6		It was found that the serum pro-inflammatory CK levels in patients with FEP increased significantly after drug treatment. This provides evidence for pro-inflammatory immune signs in SCZ.	[15]
Al-Hakeim, H. K. et al., 2015, Iraq	Serum	IL-6, IL-18, and TNFα		The study concluded that the immune response was significantly stimulated in the SCZ group. Due to the presence of heightened pro-inflammatory CKs, SCZ may be considered an inflammatory disease.	[35]
Petrikis, P. et al., 2015, Greece	Serum	IL-2 and IL-6		IL-6- and IL-2-mediated inflammatory responses may contribute to the pathogenesis of SCZ.	[36]
Lv, M. H. et al., 2015, China	Serum		TNF-α	The PANSS exhibited a notable association with TNF-α, suggesting a potential association between immune dysregulation and the manifestation of psychopathological symptoms in individuals with SCZ, as well as cognitive impairment.	[43]
El Kissi, Y. et al., 2015, Tunisia	Serum	IL-17		SANS was negatively correlated with IL-17 and positively correlated with IFN-γ. This study suggests that IL-17 may be a valuable biomarker for SCZ.	[47]
Borovcanin, M. et al., 2015, Serbia	Serum	IL-23		Elevated IL-23 levels in patients with psychosis, irrespective of antipsychotic treatment, appear to serve as an inherent biomarker for the disease.	[50]
Goldsmith, D. R. et al., 2016, USA	Blood	IL-1RA, TNF-α, sIL-2R, and IL-6		The treatment of the disease leads to a reduction in pro-inflammatory CKs and elevated anti-inflammatory CKs.	[41]
Li, H. et al., 2016, China	Plasma	IL-17, IL-23, and TGF-β1		IL-17 level was positively correlated with SCZ severity and aggressive behavior. Research suggested that elevated IL-17 levels may serve as a potential biomarker for both SCZ and aggressive behavior.	[49]
Hong, S. et al., 2017, USA	Plasma	MCP-1, MIP-1β, CCL11, TARC, and MDC		The linear combination of CCL11 and MDC exhibited a positive correlation with age, severity of negative symptoms, and duration of SCZ. With advancing age, interventions targeting the normalization of chemokine levels may potentially enhance the physical and mental well-being of individuals with SCZ.	[51]
Lesh, T. A. et al., 2018, USA	Plasma	IL -1β, IL-2, IL-6, and IFN-γ		The percentage of grey matter in the whole brain was negatively correlated with levels of IL-12 and IFN-γ, while the thickness of the prefrontal cortex showed a positive correlation with IL-12 and IFN-γ. Additionally, the IL-1β level was positively associated with psychotic symptoms in SCZ.	[37]
Dahan, S. et al., 2018, Israel	Serum	IL-8, IL-2R, and IL-6		The PANSS score exhibited a positive correlation with the levels of IL-6 and IL-2R.	[27]
Gallego, J.A. et al., 2018, USA	CSF	IL-1β, IL-6, and IL-8,		Subgroup analyses of IL-6 revealed that antipsychotics had no significant effect on IL-6 levels but were associated with increased IL-6 values in the early stages of the disease.	[28]
Xu, L. et al., 2018, China	Peripheral blood mononuclear cells	IL-8		The involvement of IL-8 in the pathophysiological changes observed in SCZ appears to be specific. Furthermore, these findings offer novel evidence supporting the autoimmune hypothesis of SCZ.	[31]
Quidé, Y. et al., 2019, Australia	Serum	IL-6 and TNF-α		The distinct association between exposure to trauma and levels of pro-inflammatory CKs across various diagnostic categories implies that trauma may exert diverse effects on stress and the immune system within these patient populations.	[38]
Ermakov, E.A. et al., 2023, Russia	CSF	IL-8		Dysregulation of chemokine expression may contribute to the development of neuroinflammation in individuals with SCZ.	[32]
	Blood	IL-8, MIP-1β, MCP-1, and CCL11			
Halstead, S. et al., 2023, Australia		IL-2, IL-1β, IL-8, TNF-α, IFN-γ, and IL-6	IL-12	Individuals with SCZ spectrum disorder have baseline levels of altered inflammatory proteins throughout the course of the disease, which are reflected in persistently elevated pro-inflammatory proteins, while patients with acute psychosis may have superimposed immune activity.	[33]
Patlola, S. R. et al., 2023, Ireland	Plasma	IL-1β, IL-6, and TNF-α		Among patients with SCZ, a significant inverse correlation was observed between cognitive performance in five domains (executive function, attention processing speed, verbal and visual learning and memory, working memory) and systemic plasma levels of TNF-α, IL-1β, and IL-6.	[39]

Abbreviations: IL, interleukin; MDC, macrophage-derived chemokine; TNF, tumor necrosis factor; CSF, cerebrospinal fluid; INF, interferon; MIP, macrophage inflammatory protein; MCP-1, monocyte chemoattractant protein-1; TGF, transforming growth factor; CCL11, categorization protein-11; sIL-2R, interleukin-2 receptor; MIP-1β, macrophage inflammatory protein-1β; TARC, thymus- and activation-regulated chemokine.

**Table 2:** Summary of the studies that link schizophrenia and anti-inflammatory cytokines.

Author, Year and Country	Tissue	Upregulated	Downregulated	Main Finding	Reference(s)
Potvin, S., 2008, Canada	Peripheral blood leukocytes	IL-1RA		IL-1RA is produced primarily by innate immune cells, indicating primary alterations in the SCZ immune system.	[34]
Kunz, M., 2011, Brazil	Serum	IL-10		The results showed chronic immune activation of SCZ.	[52]
Xiu, M.H., 2014, China	Serum		IL-10	Decreased IL-10 may be linked to negative symptoms of SCZ and cognitive impairment.	[53]
Li, H., 2016, China	Plasma	TGF-β1		TGF-β1 levels were positively correlated with PANSS.	[49]
Goldsmith, D.R., 2016, USA	Blood	IL-1Ra		The heterogeneity of IL-1RA was not significant.	[41]
Fu, G., 2019, China	Venous blood	IL-10		Elevated levels of IL-10 are associated with a breakdown of the integrity of the microstructure of white matter in SCZ. This evidence suggests that inflammation can regulate the pathological microstructure of white matter and is closely associated with SCZ.	[54]
Zhou, Y., 2019; China	Plasma	IL-1Ra		IL-1Ra may play an important role in the etiology of SCZ.	[44]
Mednova, I.A., 2022, Russia	Serum	IL-10		In a SCZ course of 10 years or more, IL-10 levels were higher than those in a SCZ course of 5 years or less. CK imbalances in individuals with SCZ are revealed, depending on the gender and clinical features of the disease.	[55]
Halstead, S., 2023, Australia	—	IL-10 and IL-1Ra	IL-4	Study quality and methodological, demographic, and diagnostic factors for most of the assessments did not significantly affect the results for most inflammatory markers.	[33]

Abbreviations: IL, interleukin; TGF, transforming growth factor.

**Table 3:** Summary of the studies that link schizophrenia and other cytokines.

Author, Year and Country	Tissue	Upregulated	Downregulated	Main Finding	Reference(s)
Bresee, C., 2009, USA	Serum	sIL-2R		Elevated SIL-2R levels are associated with etiology.	[56]
Fernandez-Egea, E., 2013, UK	Plasma	CCL11		CCL11 may be involved in the harmful effects of cannabis on brain function and mental health disorders.	[60]
Xiu, M.H., 2015. China	Serum	IL-3		There was a positive correlation between IL-3 level and PANSS. The findings suggested that the IL-3 pathway is related to the etiology of SCZ.	[57]
Fu, Y.Y., 2016, China	Serum	IL-3		Reduced IL-3 levels in SCZ suggest that immunosuppression may be associated with the development of SCZ.	[58]
Chase, K.A., 2016, USA	Peripheral lymphocytes			CBRs mediate cytokine release, with dysregulated CK levels demonstrated in individuals with SCZ.	[61]

Abbreviations: sIL-2R, interleukin-2 receptor; IL, interleukin; CCL11, categorization protein-11.

**Table 4:** Study summary on the efficacy of anti-inflammatory drugs.

Antipsychotics.	Effects	Cytokines	References
Fingolimod	Neuroprotective and anti-inflammatory	—	[67]
Berberine	Improves negative symptoms through anti-inflammatory effects	CRP ↓	[68]
PEA and risperidone	Safe relief of primary negative symptoms	—	[69]
Risperidone	Anti-inflammatory effect	TNF-α ↓, IL-6 ↓, IL-1β ↓,	[70]
Adalimumab	Effective in the treatment of negative and general psychopathological symptoms without side effects	IL-8 ↓, CRP ↓, TNF-α ↓, IL-6 ↓, and IL-1β ↓	[71]
Minocycline	Anti-inflammatory and neuroprotective	TNF-α ↓, IL-1β ↓, and IL-6 ↓	[72]
Risperidone	Anti-inflammatory and neuroprotective	IL-6 ↓ and TNF-α ↓	[72]
Clozapine	Clozapine exhibited a more pronounced impact on immune function in female patients diagnosed with SCZ	IL-2 ↓	[73]

Note: ↓: cytokine levels decreased. Abbreviations: SCZ, schizophrenia; IL, interleukin; TNF, tumor necrosis factor; CRP, C-reactive protein; PEA, palmitoyl ethanol amide.

## Abbreviations

SCZ	Schizophrenia
CNS	Central nervous system
CSF	Cerebrospinal fluid
CKs	Cytokines
EVs	Extracellular vesicles
INF	Interferon
TNF	Tumor necrosis factor
FEP	First episode of psychosis
IL	Interleukin
TGF	Transforming growth factor
MIP	Macrophage inflammatory protein
NMDAR	N-methyl-D-aspartate receptor
DAR	Dopamine receptor
CRP	C-reactive protein
sIL-2R	Interleukin-2 receptor
HCs	Healthy controls
CCL11	Categorization protein-11
MCP-1	Monocyte chemoattractant protein-1
MIP-1β	Macrophage inflammatory protein-1β
TARC	Thymus- and activation-regulated chemokine
MDC	Macrophage-derived chemokine
BDNF	Brain-derived neurotrophic factor
PEA	Palmitoyl ethanol amide
MSC-exo	MSC-derived exosomes
BBB	Brain–blood barrier
MSC	Mesenchymal stem cell
